# Recurrent appendicitis following successful conservative management of an appendicular mass in association with a foreign body: a case report

**DOI:** 10.4076/1757-1626-2-7776

**Published:** 2009-07-24

**Authors:** Sudeshna Sar, Kamal K Mahawar, Ralph Marsh, Peter K Small

**Affiliations:** 1 Department of General Surgery, Sunderland Royal HospitalKayll Road, Sunderland SR4 7TPUK; 2 Department of Radiology, Sunderland Royal HospitalKayll Road, Sunderland SR4 7TPUK

## Abstract

**Introduction:**

Interval appendicectomy is not routinely indicated after successful resolution of an appendix mass. Whether this policy can also be applied to patients with appendicular foreign body presenting with an appendix mass remains a matter of debate. We report here a patient who presented with recurrent symptoms following conservative management of an appendicular mass associated with a foreign body in the appendix. We also review the available literature briefly.

**Case presentation:**

A 70 year old gentleman was admitted with right iliac fossa pain, tenderness, and raised inflammatory markers. A computed tomography scan of his abdomen showed an appendix mass with a small abscess and a linear opaque foreign body. His symptoms resolved completely on conservative management with intravenous antibiotics. A colonoscopy few weeks later was unremarkable. He presented with recurrent symptoms a few months later. A repeat computed tomography scan showed persistent appendicular abscess with the same foreign body in it. A laparotomy with appendicectomy, abscess drainage and removal of the foreign body was carried out with satisfactory outcome.

**Conclusion:**

Surgeons should be aware of appendicular foreign body as a cause of persistent/recurrent symptoms after conservative management of appendicular mass. These patients require prompt surgery and formal appendicectomy. Interval appendicectomy should be considered.

## Case presentation

**Figure 1.**
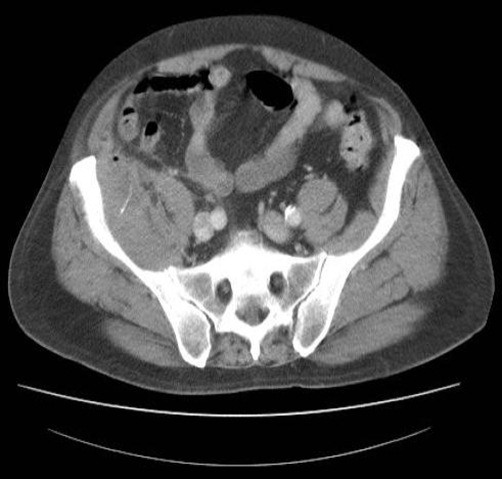
CT scan of the abdomen showing an appendix mass with a small abscess and a linear opaque foreign body.

A 70 year old, white, British male patient was admitted as an emergency with right iliac fossa pain, tenderness, and raised inflammatory markers. A computed tomography scan of his abdomen showed an appendix mass with a small abscess and a linear opaque foreign body in it (Figure 1). His symptoms subsided completely on conservative management with intravenous antibiotics. Colonoscopy two months later did not show anything untoward. At a follow up review in surgical clinic, appendicectomy was not deemed necessary and the patient was discharged from further follow up.

**Figure 2.**
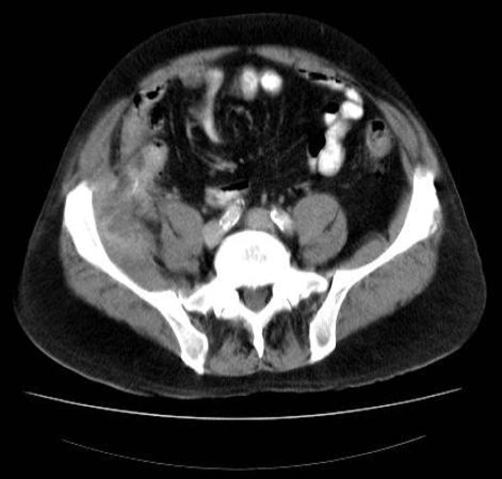
Repeat CT scan of the abdomen a few months later showing the same foreign body embedded in iliopsoas muscle with an abscess in the area.

**Figure 3.**
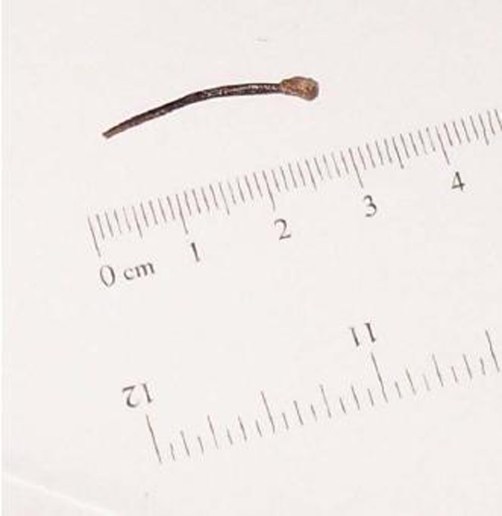
The foreign body removed from the abscess cavity at the time of surgery.

The patient presented again to us a few months later with recurrent symptoms in the right iliac fossa and raised inflammatory markers. A repeat computed tomography scan of the abdomen showed a persistent abscess in the area with the same foreign body which had migrated into the iliopsoas muscle (Figure 2). The patient underwent appendicectomy, drainage of abscess and removal of the foreign body (Figure 3). He made a satisfactory postoperative recovery and was well at the time of his clinic appointment 3 months after the surgery.

## Discussion

Foreign bodies are a rare cause for appendicitis [1,2]. The first case was recorded in the 18^th^ century when in 1736, Claudius Amyand, surgeon in the Westminster Hospital, London operated on an 11 year old boy who had a persistent faecal fistula in the right scrotal hernia. During surgery the appendix was found to be perforated by a pin [3]. In 1971, Balch and Silver reported on 7 foreign bodies in the appendix after reviewing approx 13228 cases of appendicectomies [4]. A review of 71,000 appendicectomies by Collins showed that 51.8% contained a foreign body, of which only 5.5% were unusual foreign bodies. The rest were parasitic worms or faecoliths [5,6].

Risk factors include sharp, thin, stiff, long objects and objects weighting greater than the bowel fluid content thus enabling them to arrest in the caecum and gravitate towards its dependent position [4,7]. A variety of foreign bodies have been found in the appendix ranging from sewing needles, retained shot pellets, tongue studs, endodontic files, drill bits, dog hair, toothbrush bristle, toothpicks , fishing lines, mercury (after ingestion of the bulb of a thermometer) and condom fragments [4,6,8-12]. Complications usually depend on the size and shape of the object. Elongated, sharp objects which account for 75% of foreign bodies in the appendix are more likely to cause perforations, appendicular abscesses and peritonitis. Blunt objects, which account for <12% of all the foreign bodies can become coated with faecal coating, enlarge and fully or partially obstruct the appendicular lumen, resulting in appendicular mucocoele or decubital perforation [4,5,10,13].

Presentation can vary from asymptomatic to abdominal pain, with or without vomiting or diarrhoea. A history of recent ingestion of the foreign body is sometimes obtained. On examination low grade pyrexia is often seen with associated tenderness/peritonism in the right iliac fossa or lower abdomen. White cell counts and C reactive protein may be raised depending upon the degree of inflammation. Radio opaque foreign bodies are readily visualised in the right lower quadrant. Free intraperitoneal air is seen if the object has caused perforation of the gastrointestinal tract. A computed tomography scan would usually be required to confirm the diagnosis.

A symptomatic appendicular foreign body will need an appendicectomy. Most patients present with features of appendicitis and undergo an appendicectomy. However in some high risk patients with diagnostic difficulties or those presenting with appendix mass, a non operative approach may be adopted initially. After successful non operative management of appendicitis, interval appendicectomy is not routinely necessary and can safely be omitted [14]. As far as we are aware there is no available study in the published English medical literature evaluating this approach in patients with appendicular foreign body presenting with an appendix mass. In our patient the initial non operative approach had to be abandoned in favour of surgery.

## Conclusion

Surgeons should be aware of appendicular foreign body as a cause of appendicitis/appendix mass. This can present as a cause of persistent/recurrent symptoms after conservative management of appendicular mass. These patients require prompt surgery and formal appendicectomy. Whether these patients should undergo routine interval appendicectomy remains to be investigated.
